# Tarsoaponeurectomy as an alternative in difficult blepharoptosis cases

**DOI:** 10.1186/s12886-016-0208-2

**Published:** 2016-03-31

**Authors:** Selam Yekta Sendul, Burcu Dirim, Mehmet Demir, Zeynep Acar, Atilla Gokce Demir, Ali Olgun, Semra Tiryaki, Cemile Ucgul, Dilek Guven

**Affiliations:** Department of Ophthalmology, Sisli Hamidiye Etfal Training and Research Hospital, Etfal Street 34280, Sisli, Istanbul Turkey; Department of Ophthalmology, Ulucanlar Eye Training and Research Hospital, Ulucanlar street, 06030 Altındag Ankara, Turkey

## Abstract

**Background:**

The purpose of this study was to evaluate the results of tarsoaponeurectomy in patients with unsuccessful results after repetitive surgery or who developed post-traumatic blepharoptosis.

**Methods:**

The files of 107 patients (136 eyes) on whom surgery was performed between January 2010 and December 2014 due to blepharoptosis were scanned retrospectively. Among these patients, the files and operational notes of eight patients who underwent surgery through the method of tarsoaponeurectomy were examined in detail. The epidemiological data, indication for surgery, previous ptosis and/or eyelid surgeries and trauma histories, preoperative and postoperative measurement data (palpebral space (PS), margin reflex distance (MRD1, MRD2), levator muscle function (LMF)) of the patients were recorded. The follow-up time of the patients was 7 to 34 months with an average of 16 months.

**Results:**

A total of eight patients consisting of three females and five males were included in the study. The age range was 19 to 63 years with an average of 39 ± 16.2 years. Four patients had traumatic ptosis history whereas four patients had previous multiple levator procedure surgery history. Those patients with a history of ptosis had undergone surgery with levator procedure at least two times. Additionally, one patient had upper eyelid entropion, one had anophthalmic socket syndrome, and one had exposure keratopathy and traumatic dilated pupil. Seven patients had ptosis in the left eye whereas one patient had ptosis in the right eye. All patients were given a tarsoaponeurectomy as the basic surgical procedure while the patient with entropion was given a tarsal fracture and ear cartilage grafting as additional surgery. Two patients with vertical notching were also given a vertical blepharotomy through which a strip of tarsus was removed.

**Conclusions:**

Tarsoaponeurectomy is an alternative method for oculoplastic surgeons used to deal with patients on whom sufficient and desired results have not been achieved despite repetitive surgery and in post-traumatic cases where levator muscle and aponeurosis cannot be dissociated peroperatively.

## Background

Blepharoptosis can be conventionally classified as congenital or acquired. Acquired blepharoptosis can be sub-classified as myogenic, neurogenic, aponeurotic and mechanical or traumatic [1]. In the treatment of blepharoptosis, while the surgical treatment alternatives essentially vary on the basis of levator muscle function, many surgical intervention methods have been defined and discussed [1–4]. While there are several fundamental success criteria, the most important success criterion is postoperative patient satisfaction [4]. Some blepharoptosis cases constitute a difficult patient group in oculoplastic practice in terms of the results obtained. Particularly those patients who had previously undergone eyelid surgery due to various reasons, those whose initial surgical treatment had not been conducted by experienced oculoplastic surgeons and those cases with no success despite repetitive surgeries as well as those patients with eyelid anatomy disorders such as levator, tarsal and septum disorders as a result of multiple traumas may be included in this patient group.

In this study, we shall discuss the results achieved through the tarsoaponeurectomy technique in acquired ptosis cases with whom the desired results could not be achieved despite repetitive surgeries and in post-traumatic ptosis cases.

## Methods

In our clinic, the files of 107 patients (136 eyes) who underwent surgery due to blepharoptosis between January 2010 and December 2014 were scanned retrospectively. Forty five patients (58 eyes) out of 107 had been given frontalis sling surgery due to congenital or levator muscle function weakness. The remaining 62 patients (78 eyes) had been operated on using a levator procedure with different methods. Out of the latter, the files and operational notes of eight patients who had undergone a tarsoaponeurectomy operation were examined in detail. The epidemiological data, indication for surgery, previous ptosis and/or eyelid surgeries, trauma histories, preoperative and postoperative measurement data (palpebral space (PS), margin reflex distance (MRD1, MRD2), levator muscle function (LMF)) of the patients were recorded. The tarsoaponeurectomy indication was made preoperatively taking into consideration the previous multiple ptosis surgeries the patient had undergone, traumatic eyelid deformities, level of preoperative levator muscle function and damages on the tarsus in the upper eyelid. Upon reaching the levator aponeurosis and muscle peroperatively, a further evaluation was made and the decision to perform a tarsoaponeurectomy on patients who were considered as showing a low chance of success through the levator procedure was finalized.

**Fig. 1:**
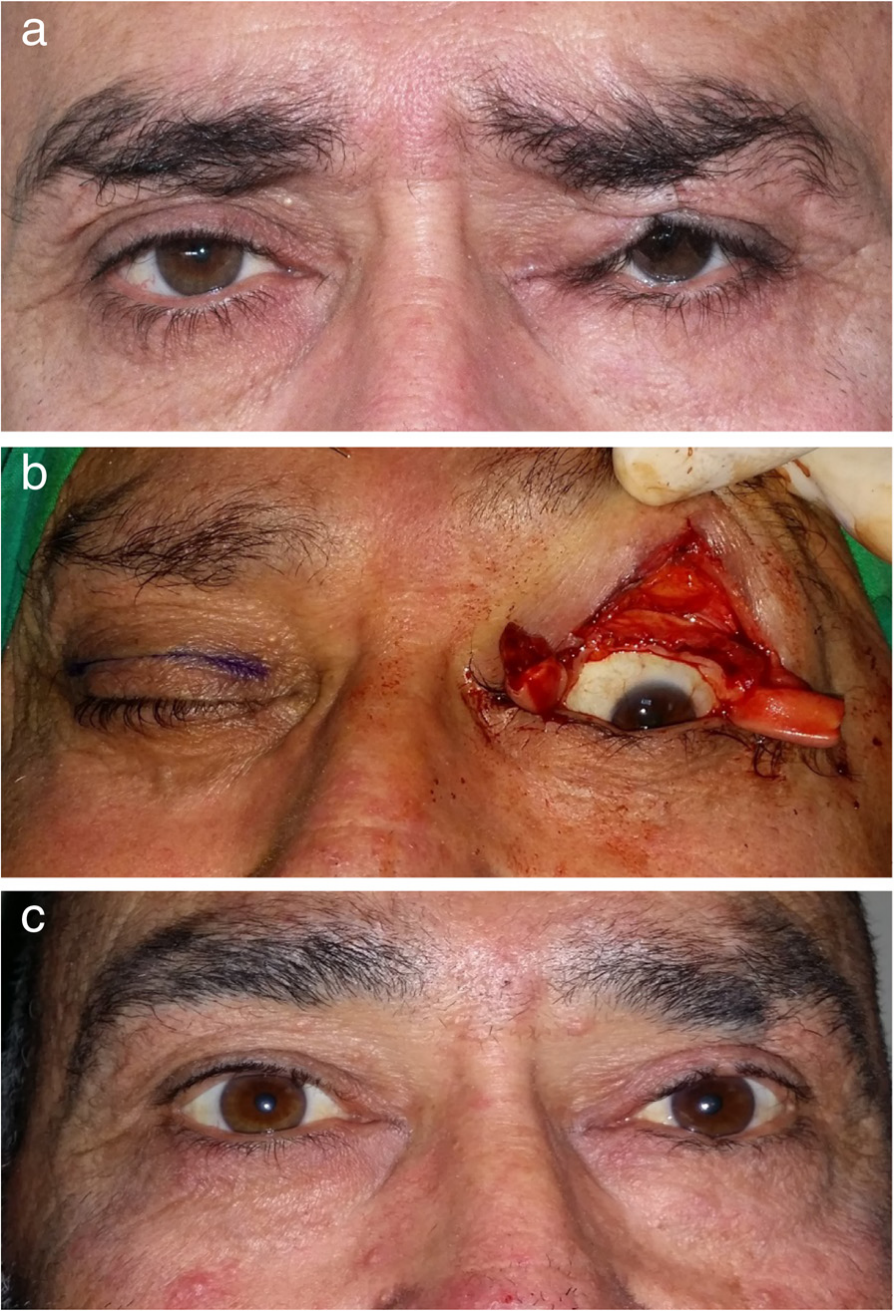
**a** 54-year-old male patient. History of vehicle accident 30 years ago. Ptosis in the left eye and notching in the eyelid are observed. **b** The patient underwent peroperative tissue adhesion removal. Then, vertical tarsectomy was performed by vertical blepharotomy as a thin strip followed by tarsoaponeurectomy. **c** Eyelid view of the same patient at postoperative year 2

The measured palpebral space (PS), levator muscle function (LMF) and MRD1 values and the postoperative complications of all patients were recorded preoperatively and postoperatively. Again, the recorded preoperative and postoperative eyelid deformations and eyelid contour disorders were examined and photographed (Fig. 1a, b, c). All patients were contacted and final follow-up examinations were made. The follow-up time of the patients was 7 to 34 months with an average of 16 months. The study was conducted in accordance with the tenets of the Declaration of Helsinki by obtaining written consent from all patients, with the approval of the local ethical committee.

### Surgical technique

**Fig. 2:**
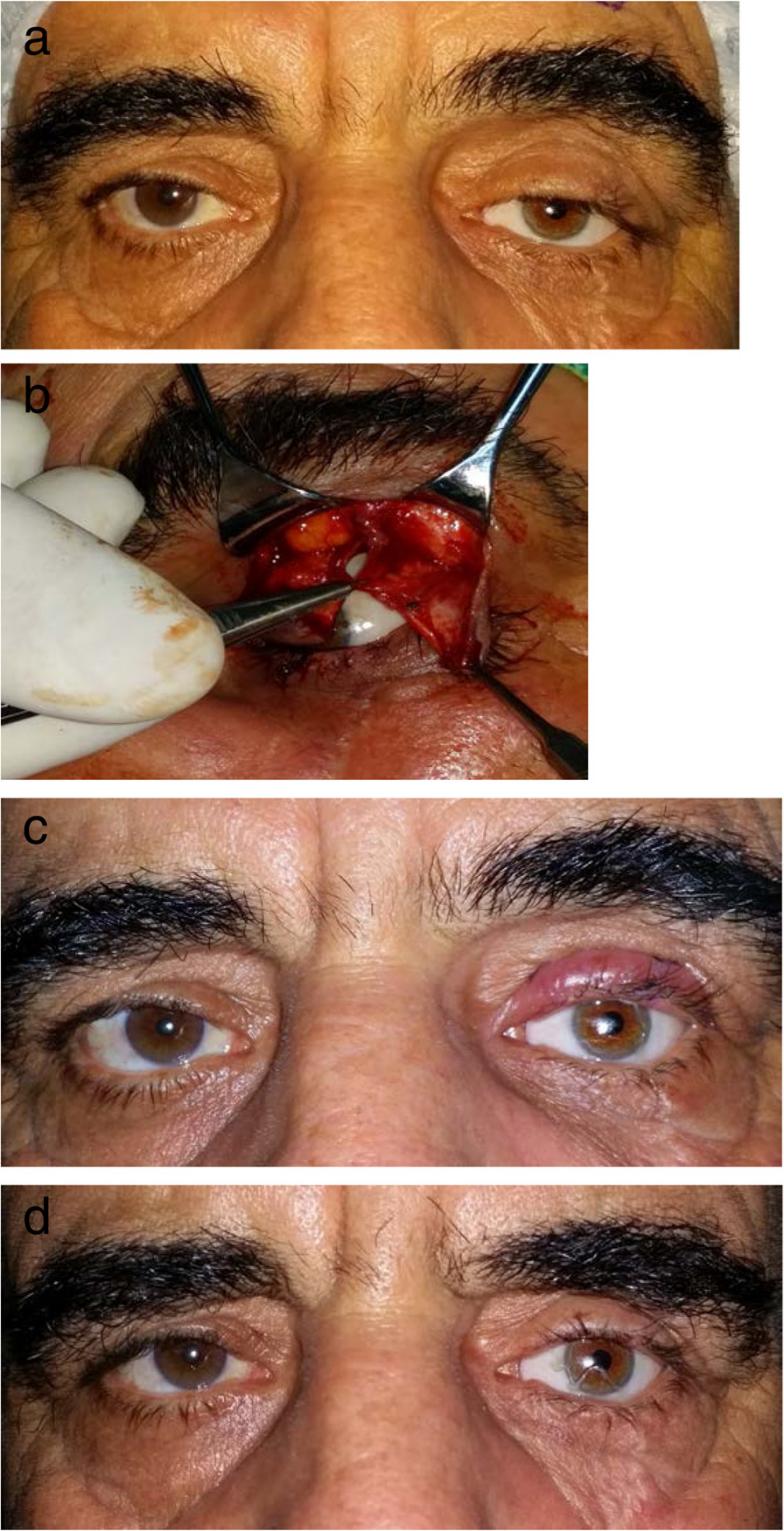
**a** 63-year-old male patient. History of sharp object injury 10 years ago. Prosthesis in the left eye due to anophthalmic socket. Distinct ptosis and notching at the temporal are observed. Even more distinct ptosis in the nasal region of the right eye is apparent. **b** Peroperative view of the same patient. Vertical tarsectomy was performed by vertical blepharotomy as a thin strip followed by horizontal tarsoaponeurectomy. **c** View at postoperative week 1. Ptosis in the right eye continues. **d** View at postoperative year 1.5 following right levator advancement

The incision line was marked with a pen preoperatively based on the eyelid sulcus line of the healthy eye. Jetokain (Lidocaine HCL 20 mm/ml+Epinephrine HCL 0.0125 mg/ml) was injected into the eyelid locally in order to reduce peroperative bleeding and all patients were operated under local anesthesia. Then, the marked section was incised and the cutaneous and subcutaneous tissues were passed. The upper eyelid tarsal tissue was reached through vertical dissection. The adhesions due to previous trauma and/or surgery were released through blunt and sharp dissection. As the septum was in a damaged state due to previous surgical treatments and/or trauma, the levator aponeurosis and muscle were reached using the preaponeurotic fat tissue as an indicator. At this stage, those patients with intact levator muscles and aponeurosis were given conventional aponeurosis surgery whereas it was decided to perform tarsoaponeurectomy to those patients who did not have their muscles, aponeurosis or tarsus intact. At this stage, the amount of tarsus and aponeurosis complex (aponeurosis, Müller muscle, conjunctiva) to be excised was determined on the basis of preoperative measurements and in comparison with the peroperative eyelid level of the other eye. An average of 1–3 mm tarsal tissue and 1–5 mm aponeurosis complex were excised. Afterwards, the aponeurosis complex was sutured to the upper edge of the tarsus with three 6/0 vicryl sutures as central, nasal and temporal. At this stage, the eyelid contour and level were compared to the healthy eyelid of the patient as a preoperative check upon which middle sutures were made. Then, three sutures forming the upper eyelid crease were located in a way as to pass through the aponeurosis complex and the skin was closed with 6/0 prolene (polyproylene) suture. After surgery contact lenses were applied to all patients for 1 week in order to prevent postoperative corneal erosion (Fig. 2a, b, c, d).

### Statistical analysis

In the descriptive statistics of the data, mean and standard deviations, median and min-max values were used. The distribution of the variables was controlled by the Kolmogorov Smirnov test. In the analysis of the quantitative data, the Wilcoxon test and the matched sample t test were used. In the analyses, the SPSS 22.0 program was used.

## Results

A total of eight patients consisting of three females and five males were included in the study. The age range was 19 to 63 years with an average of 39 ± 16.2 years. Four patients had a traumatic ptosis history whereas four patients had a previous multiple levator procedure surgery history. Each patient who had had previous multiple surgeries under a levator procedure had a history of at least two and at most four operations. Additionally, one patient had upper eyelid entropion, one had anophthalmic socket syndrome, and one had exposure keratopathy and traumatic fix dilated pupil. Seven patients had ptosis in the left eye whereas one patient had ptosis in the right eye (Table 1). All patients were given tarsoaponeurectomy as the basic surgical procedure while the patient with entropion was given tarsal fracture and ear cartilage grafting as additional surgery. Two patients with traumatic vertical notching were also given vertical blepharotomy through which tarsus in the shape of a tarsal strip was removed.

**Table 1:** Epidemiologic data, preopeartive and postoperative histories and complications of the patients.(*M* male, *F* female)

Patient	Age/Type	History	Additional disease	Additional surgery	Complications
1	27/m	ptosis - 3 times	entropion	-tarsal fracture -ear cartilage graft	corneal irritation in the early term
2	42/f	ptosis - 2 times	none	Revision surgery due to postoperative temporal drooping	corneal irritation in the early term
3	51/f	extravehicular traffic accident	-notching in the lid -exposure keratopathy -lower eyelid laxity -traumatic fix dilated pupil	lateral canthal sling (in a separate session)	- corneal irritation in the early term -continuous light sensitivity
4	63/m	sharp trauma 10 years ago	- notching in the lid -anophthalmic socket syndrome	Removal of vertical tarsal strip by vertical blepharotomy	none
5	54/m	traffic accident 30 years ago	notching in the lid	Removal of vertical tarsal strip by vertical blepharotomy	corneal irritation in the early term
6	19/m	-motorcycle accident -bilateral canalicular repair and lid reconstruction	multiple former wound scars in the facial area	none	none
7	21/m	ptosis - 2 times	none	none	none
8	35/f	ptosis - 4 times	none	none	none

**Fig. 3:**
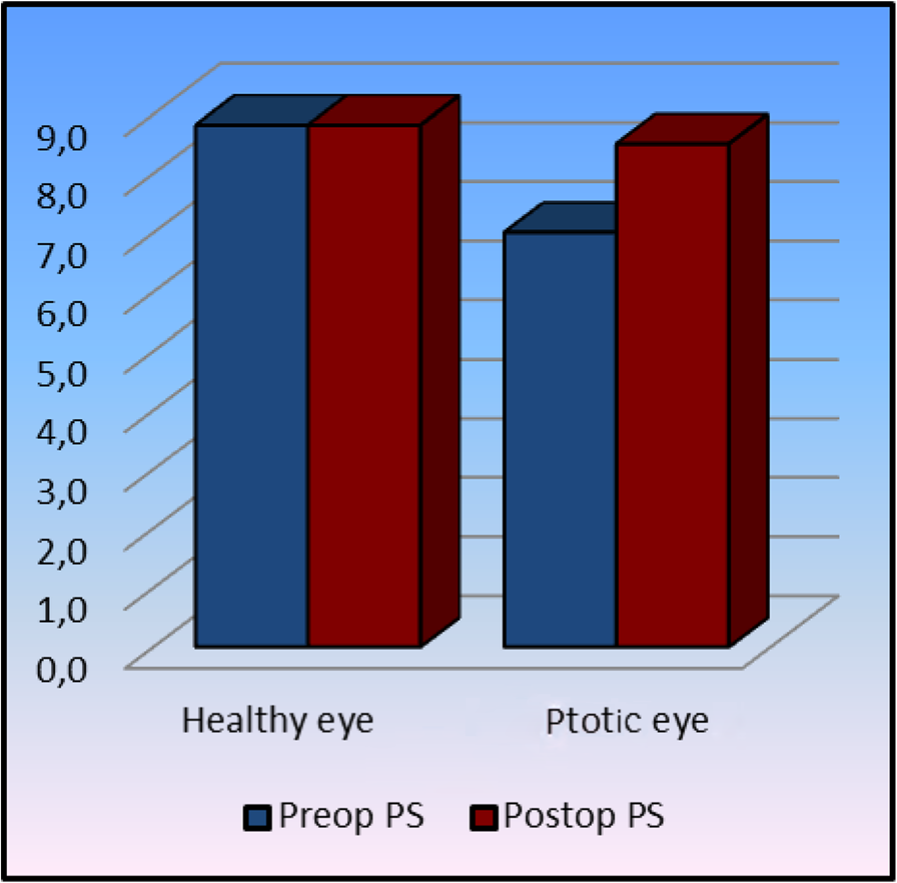
Significant increase in palpebral space is observed postoperatively in ptotic eyes

**Fig. 4:**
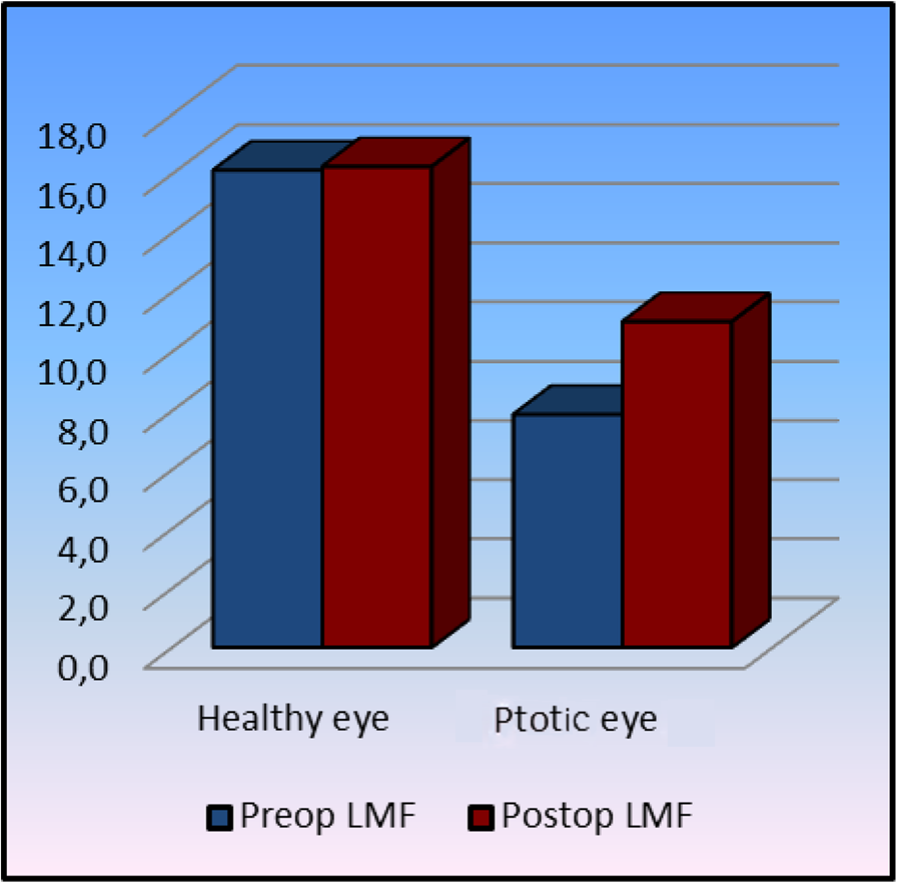
Increase in LMF is observed postoperatively in ptotic eyes, although not at statistically significant rates. (It probably occurred due to the enlargement of the movement range of the muscle following the recovery of adhesions)

**Fig. 5:**
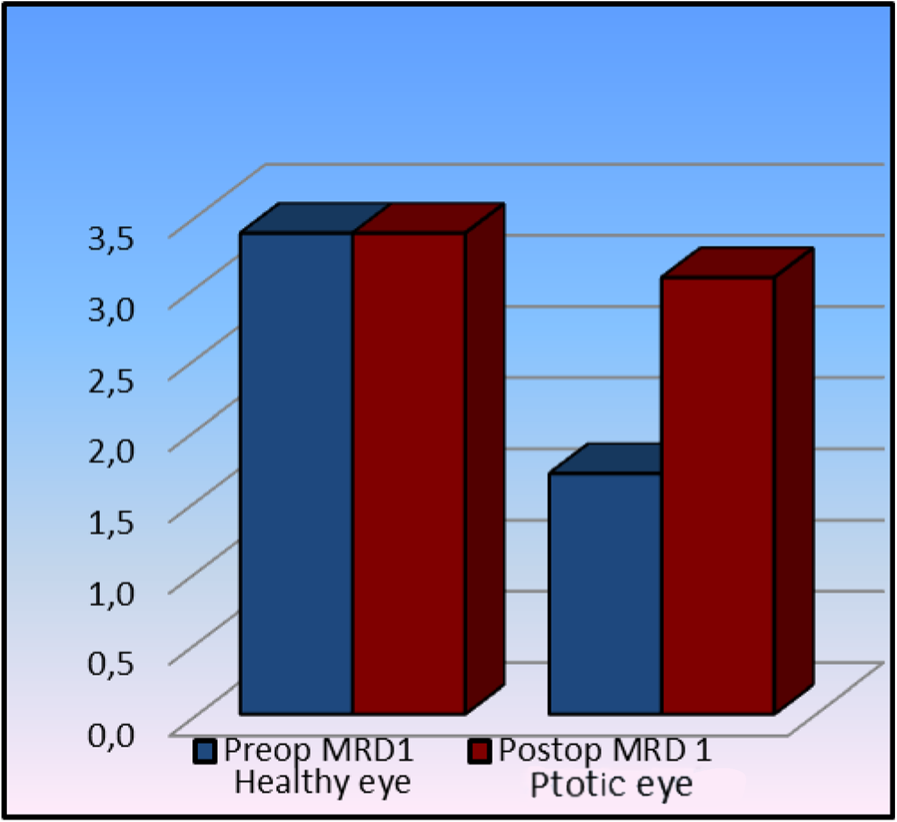
Increase in MRD1 values is observed postoperatively in ptotic eyes

The basic success criterion was taken as a quantitative ptosis measurement (Table 2). All measurements were made during clinical examination. Preoperatively, the PS value was significantly lower in the eye with ptosis compared to the normal eye (*p* <0.05) whereas postoperatively no significant difference was detected between the two eyes (*p* >0.05) and again postoperatively a significant increase was detected in the eye with ptosis compared to the preoperative values (*p* <0.05) (Fig. 3). With respect to levator muscle function, it was significantly reduced in the eye with ptosis both preoperatively and postoperatively compared to the healthy eyelid (*p* <0.05) whereas no significant difference was detected in the postoperative levator function of the unhealthy eye compared to the preoperative value (*p* <0.05) (Fig. 4). In terms of MRD1 value, the preoperative MRD1 value in the eye with ptosis was significantly lower than that of the healthy eye (*p* <0.05) while there was no significant difference between the two postoperative values (*p* >0.05). Again, in the eye with ptosis, the postoperative MRD1 value showed a significant increase compared to the preoperative value (*p* <0.05) (Fig. 5). We did not observe any serious complications such as eyelid instability, ectropion or entropion in any of our cases. However, all cases showed temporary lagophthalmos of 1–3 mm continuing until postoperative month 1. Within this period, all patients were given intense eye-drop and gel therapy thereby preventing any ocular surface damage that might have developed due to lagophthalmos.

**Table 2:** Preoperative and postoperative statistical data

	Healthy eye			ptotic eye			*p*
	Avg. ± s.d.	Med(Min-Max)		Avg. ± s.d.	Med(Min-Max)		
Vision	1.8 ± 2.3	1.0	1-7	0.7 ± 0.4	0.7	0.0-1.0	0.068
Preop PS	8.8 ± 0.7	9.0	8-10	7.0 ± 1.4	7.0	5.0-10.0	0.004
Postop PS	8.8 ± 0.7	9.0	8-10	8.5 ± 0.5	8.5	7.5-9.0	0.305
*Preop/Postop Change*	1.000			0.030			
Preop LMF	16.1 ± 1.9	16.0	13-18	7.9 ± 4.0	7.0	2.0-15.0	0.012
Postop LMF	16.3 ± 2.0	16.5	13-18	11.0 ± 2.9	10.5	6.0-16.0	0.012
*Preop/Postop Change*	0.317			0.121			
Preop MRD1	3.4 ± 0.4	3.5	3-4	1.7 ± 1.2	1.0	1.0-4.5	0.016
Postop MRD 1	3.4 ± 0.4	3.5	3-4	3.1 ± 0.4	3.0	2.5-3.5	0.096
*Preop/Postop Change*	1.000			0.029			

Wilcoxon test/Matched sample *t* test

## Discussion

In the correction of blepharoptosis, three primary surgical methods exist. In congenital ptosis cases and/or in ptosis cases with poor levator function, frontalis sling techniques may be used whereas in acquired ptosis cases, levator resection or levator aponeurisis advancement techniques may be used depending on the strength of the levator muscle. It is possible to choose one of these methods on patients who apply for primary surgery [5–8]. However, there is a group of patients who are very difficult. This group includes those ptosis cases on whom no successful results are obtained despite repetitive surgery and also those ptosis cases that develop due to eyelid tissue damage following orbital or eyelid traumas. In this study, we evaluated the tarsoaponeurectomy method which we performed on this group of patients, the indications for the procedure, the results thereof and postoperative complications.

Tarsal resection has a long history in blepharoptosis surgery. In his presentation, Reifler [9] detailed the basic points whereas Anatole Pierre Louis Gillet de Grandmont [10] reported that correction in ptosis was obtained through partial tarsal resection. Hervoue¨t and Tessier [11] published a study in 1956 and Mustarde [12] published another in 1975 where they defined tarsectomy combined with plication of the levator aponeurosis-Müller muscle complex, i.e. “split-level” operation.

Patel SM et al. [13] reported that they received excellent results in congenital ptosis cases with poor levator muscle functions through the levator aponeurosis-Müller-conjunctiva complex surgery combined with tarsus, and regarding complications, they only detected temporary lagophthalmos and exposure keratopathy that both recover over time, however they further stated that they did not have sufficient long term information. On the other hand, Park J et al. [14], in their comparative study in which they performed super-maximum levator resection surgery alone or in combination with superior tarsectomy again in patients with poor levator muscle function, reported that the eyelid was lifted more in the latter group. They also reported that they did not identify any possible complications that may have occurred in the resection of the tarsus such as eyelid instability, ectropion, entropion, etc. In our study, all patients consisted of cases with fair or good levator muscle function, and we obtained successful results on them. Again, similar to the above mentioned studies, although temporary lagophthalmos was detected in the early postoperative term, it recovered completely by the end of month 1. Again, although we detected discomfort due to suture irritation in our patients, we overcame this problem in the early postoperative term through the use of contact lenses and also intensive use of eye drops and gels. None of the cases showed eyelid instability.

The question that comes to mind in this matter is the amount of tarsus-aponeurosis-Müller-conjunctiva complex to be resected. Of course, in this regard, the amount and degree of ptosis come into play. [13–15] Beard [15] defines eyelid drooping up to 2 mm as mild, 3 mm as moderate and 4 mm and above as severe ptosis. In this matter, Patel et al. [13] reported that they made their decisions on the basis of the level of ptosis and that they performed the Müller-conjunctiva resection about twice as many times as the tarsal resection and that in mild ptosis cases with poor levator muscle function, they performed 2–3 mm tarsus and 4–6 mm Müller-conjunctiva, in moderate ptosis cases 3–4 mm tarsus, 6–8 mm Müller-conjunctiva, and in severe ptosis cases 4–5 mm tarsus and 8–10 mm Müller-conjunctiva resection. We conducted surgery under local anesthesia and therefore we made our decisions by taking into consideration the preoperative measurement data and comparing it with the peroperative healthy eyelid level. After we performed the first resection and made the first sutures, just like the case in the levator advancement technique, we compared it with the other eyelid by giving the patient instructions and making the patient sit when necessary. At this stage, we made additional resections when insufficient corrections or contour disorders were in question. Due to the fact that the muscle function levels of our patients were fair to good, the tarsus-aponeurosis-Müller-conjunctiva complex reduced the resection amount.

Another question was as to what can be done in the case of insufficient correction or overcorrection. Park et al. [16] reported that two patients in group 2 were detected with insufficient correction, they performed the frontalis sling on one and the levator resection on the other and they performed a combined treatment on their patients who had unsuccessful results from group 1. As a matter of fact, this was the question that worried us most during surgery in that we had little chance as to what we could do in the case of overcorrection. Therefore, we performed the surgery by keeping the resection amount at low levels peroperatively and constantly comparing it with the other eyelid. In our study, only one patient developed temporal eyelid drooping and we corrected this case through a second surgery by simply removing some conjunctiva-müller-aponeurosis complex from the temporal area. According to us, the major disadvantage of this surgery is that there are no alternatives other than placing graft to the posterior lamellar area when overcorrection happens. Thankfully, we did not come across any postoperative overcorrections in any of our patients.

Another question to be examined is what are the long term results of this surgery. Unfortunately, there is no sufficient study or data in the literature in this regard. According to our experience of about 3 years of follow-ups, insufficient corrections are revealed at the end of postoperative month 1. We did not detect any changes in our patients in the subsequent follow-ups. However, it is not proper to reach a final judgment in this regard due to the low number of patients.

## Conclusions

In conclusion, with regard to the tarsoaponeurectomy method, we submit the following points: 1. Tarsoaponeurectomy should never be the initial choice in patients with good levator muscle function; 2. The tissue to be resected should be kept minimal during peroperative resection because, in case of overcorrection, there is no other alternative but placing a new graft; and 3. Tarsoaponeurectomy is an alternative method for oculoplastic surgeons to be used only for those patients in whom sufficient and desired results are not obtained despite repetitive surgery and in post traumatic cases where levator muscle and aponeurosis cannot be dissociated peroperatively.

### Ethics approval and consent

This study was approved by Sisli Hamidiye Etfal Training and Research Hospital Local Ethical Committee. Further, written consent was obtained from the patients shown in the figures. (Approval date:01.09.2015, Approval number:1063).

## Abbreviations

PS: palpebral space
MRD1: margin reflex distance1
MRD2: margin reflex distance2
LMF: levator muscle function
